# Synergistic effects of nitrogen-doped carbon and praseodymium oxide in electrochemical water splitting

**DOI:** 10.1038/s41598-023-43774-8

**Published:** 2023-10-30

**Authors:** Patrycja Grabowska, Mariusz Szkoda, Malgorzata Skorupska, Jerzy P. Lukaszewicz, Anna Ilnicka

**Affiliations:** 1 Faculty of Chemistry, Nicolaus Copernicus University in Torun, Gagarina 7, 87-100 Torun, Poland; 2 Department of Chemistry and Technology of Functional Materials, Faculty of Chemistry, Gdańsk University of Technology, Narutowicza 11/12, 80-233 Gdańsk, Poland; 3 Advanced Materials Center, Gdańsk University of Technology, Narutowicza 11/12, 80-233 Gdańsk, Poland; 4 Centre for Modern Interdisciplinary Technologies, Nicolaus Copernicus University in Torun, Wilenska 4, 87-100 Torun, Poland

**Keywords:** Chemistry, Energy science and technology, Materials science

## Abstract

Hybrid materials featuring perovskite-type metal oxide in conjunction with heteroatom-doped graphene hold immense promise as alternatives to costly noble metal catalysts for electrochemical water splitting, facilitating the generation of environmentally friendly hydrogen. In this study, perovskite-type oxide containing praseodymium, barium, strontium, cobalt, and iron atoms dispersed in a carbon matrix as a catalyst is synthesized via annealing of the carbon material with substrates for the preparation of perovskite oxide. The mass ratio of reagents regulates the porous structure and elemental composition. The result of the hydrogen evolution reaction (HER), suggests that the hybrid catalysts exhibit intermediate HER kinetics compared to the commercial Pt/C and the catalyst without carbon. The Tafel slope for HER is lower for materials containing carbon, because of the improved reaction kinetics, facilitated proton transfer, and enhanced electrochemical surface area. Therefore, the study provides an effective strategy for the preparation of catalyst and their use as the active catalyst of water splitting.

## Introduction

Electrocatalytic water splitting is considered the most promising technology for producing green hydrogen. Based on the current state of knowledge, usually, the catalytic properties in water splitting come from noble metals and their alloys and oxides. So far, platinum is the most efficient electrocatalyst for hydrogen evolution reactions^1,2^, iridium oxide^3^, and ruthenium oxide^4^ are reported to be effective catalysts for oxygen evolution reactions. However, for economic and environmental reasons, noble metal-based catalysts require advanced and expensive recycling. Therefore, it is necessary to develop noble metal-free electrocatalysts with excellent bi-functional catalytic performance in both hydrogen evolution and oxygen evolution reactions. Another key issue is that the catalysts used in the water splitting reaction should have good conductivity and stability, so carbon materials doped with nitrogen are considered a good catalytic material^5–7^. Carbon materials doped with nitrogen are distinguished by excellent electronic conductivity, moreover, doping with nitrogen atoms increases the stability of carbon materials^8^.

Metal oxides, especially rare earth elements are widely used as catalysts^9–11^. Various materials have been studied in the past towards improving the performance, where samarium-based and praseodymium-based perovskite have been tested in catalytic processes including carbon dioxide reforming of methane^12^, oxygen reduction reaction and oxygen evolution reaction^13^, syngas production^14^, and hydrogen evolution reaction^9^. Also, lanthanum-based^15^, barium-based^16^, europium-based^17–19^, CeO_2_^20,21^, nickel-doped CeO_2−x_^22^, and NiCeO_x_^23^ materials have been studied as efficient catalysts in oxygen evolution reaction, hydrogen evolution reaction, and also in oxygen reduction reaction in alkaline electrolyte. Despite intense research, serious challenges remain because of the lack of high-efficiency, low-cost, bi-functional anodic and cathodic electrocatalysts for boosting the sluggish oxygen evolution reaction and hydrogen evolution reaction^24–26^. Currently, praseodymium compounds are considered the most promising candidates to take on the aforementioned challenges, especially Pr_2_O_3_^17,27,28^, PrO_2_^29^_,_ PrFeO_3_^30^, and Pr_3_IrO_7_^26^. However, rational design of catalysts and their supports should be taken into consideration in the development of highly efficient and stable catalysts.

In this study, hybrid materials containing concurrently of nitrogen-doped graphene foam and perovskite-type oxides containing praseodymium were synthesized. The carbon matrix served as a foundational framework, enabling the in situ crystallization of the perovskite-type oxide. The presence of the carbon substrate and the porous structure formed in it causes the resulting perovskite-type oxide crystallites to be nanometer-sized. This proposed concept of electrode material composition aims to simultaneously and synergistically exploit the catalytic properties of perovskite-type oxide and carbon matrix in hydrogen evolution reaction and oxygen evolution reaction in alkaline electrolyte. The results demonstrate that the addition of carbon to perovskite-type oxide gave rise to enhanced electroactivity of catalysts in green hydrogen production by electrochemical water splitting, attributed to the enlarged specific surface area and synergies between the structural defects and nitrogen-doping effect. These studies underscore the potential of the proposed composite electrode material in advancing the efficiency of electrochemical water splitting for green hydrogen production. By exploiting the advantageous properties of the nitrogen-doped graphene foam and the perovskite-type oxide, this study paves the way for innovative strategies in sustainable energy conversion.

## Results and discussion

### Materials characterization

**Figure 1:**
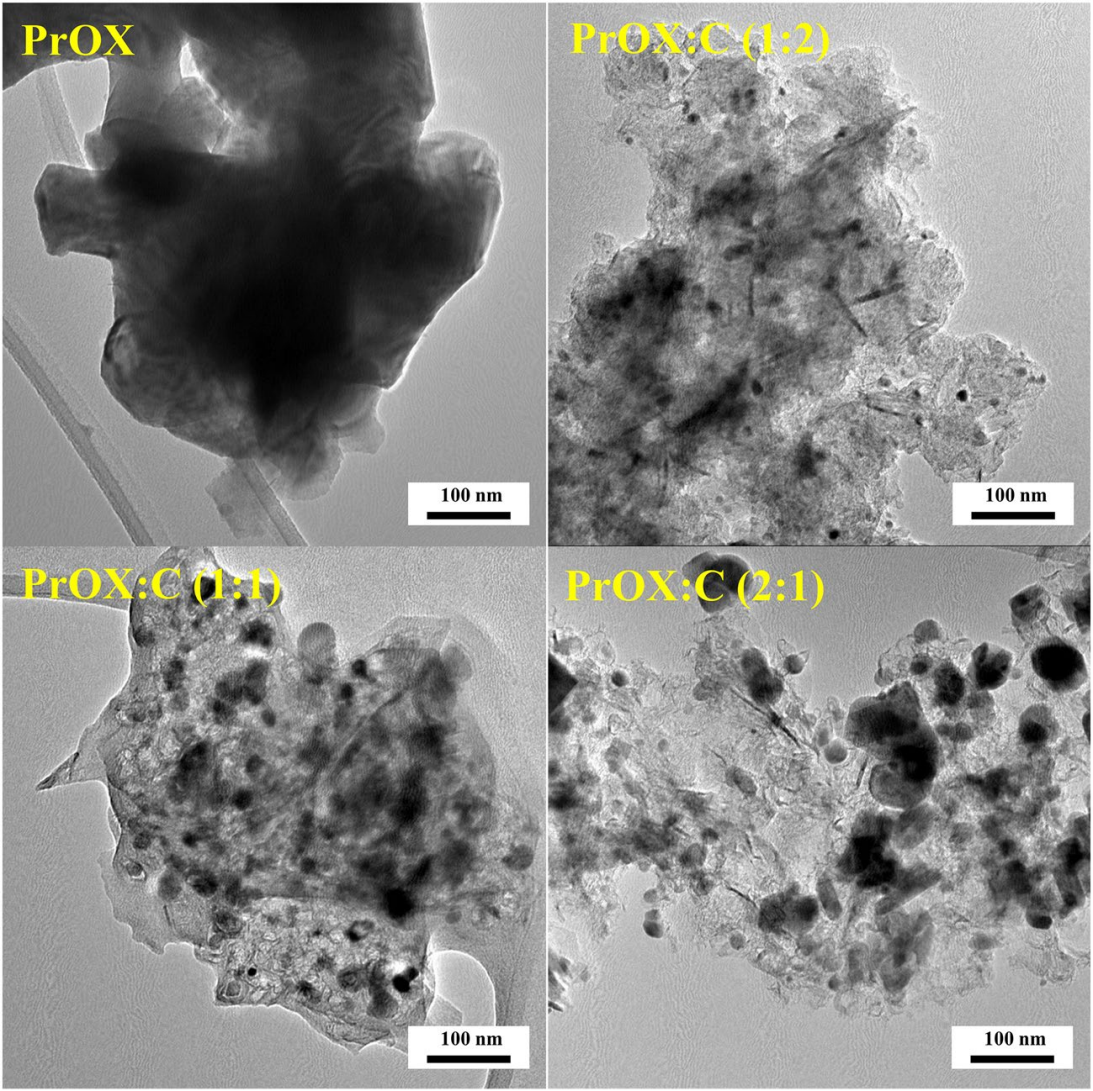
HRTEM images of pristine oxides PrOX and their hybrids with carbon of PrOX:C (1:2), PrOX:C (1:1), and PrOX:C (2:1).

The morphology of the obtained materials was determined by high-resolution transmission electron microscopy analysis. Figure 1 shows HRTEM images, confirming that the pristine PrOX oxide nanoparticles have agglomerated into larger bulk.

**Figure 2:**
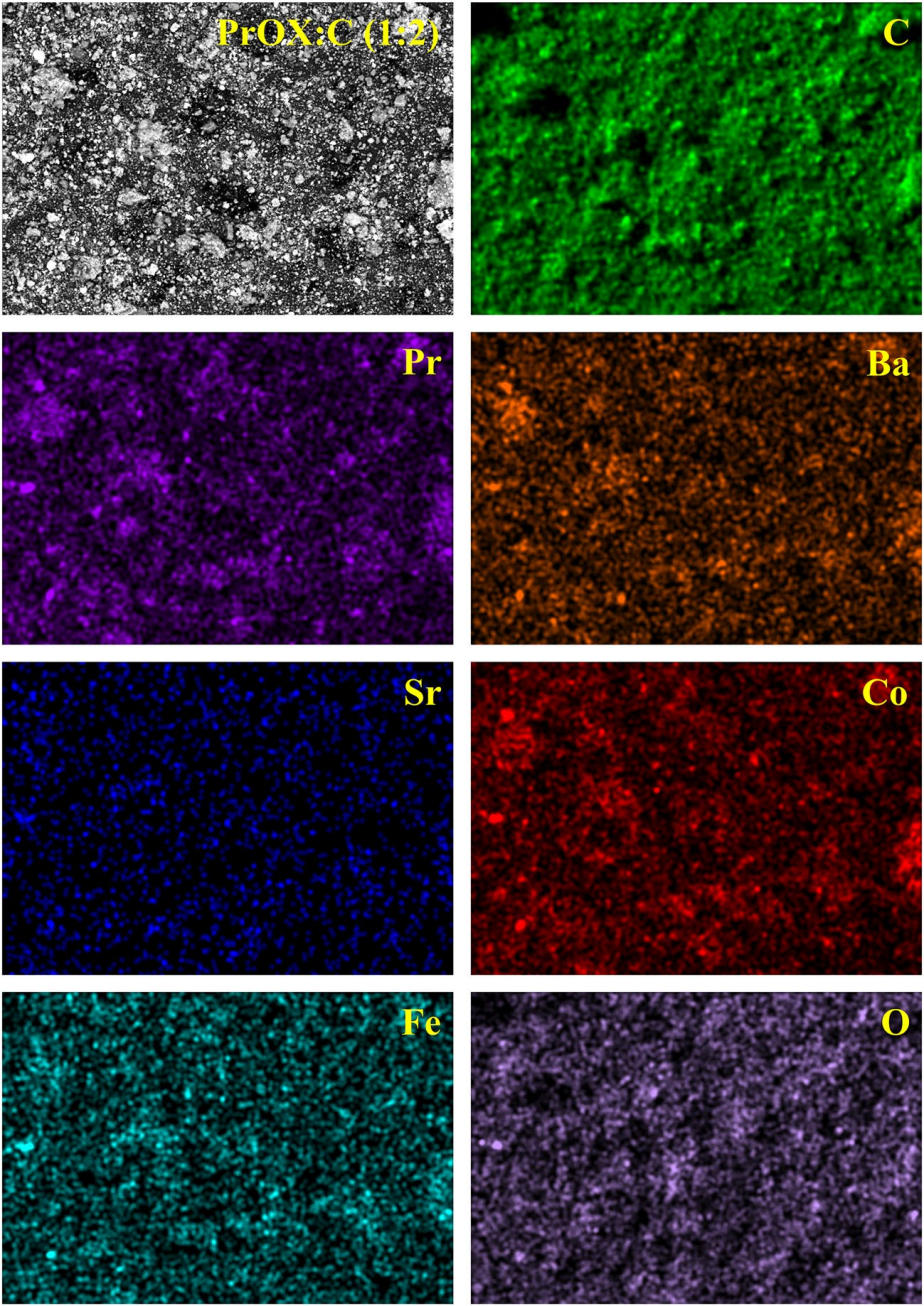
Elemental mapping for the sample PrOX:C (1:2).

PrOX is characterized by a disordered structure, but it can be seen that it is several layers overlapping, as lighter and darker spots can be distinguished. In the case of hybrid materials, even dispersion of catalyst particles in the carbon matrix can be seen. Depending on the ratio of catalyst to carbon matrix, larger catalyst agglomerates can be distinguished, in the case of PrOX:C (2:1), or smaller clusters in the case of PrOX:C (1:2) on the carbon substrate. In all PrOX:C samples, in addition to uneven catalyst agglomerates, one can also distinguish elongated structures distributed throughout the sample with particle size in the range of 10 − 80 nm. Figure S1 shows images of SEM–EDX profiles with peaks confirming the presence of Pr, Ba, Sr, Co, Fe, and O elements. The homogeneous distribution of these elements on the surface of PrOX:C (1:2) sample is presented in Fig. 2.

Also in the case of this sample on the SEM image visible small agglomerates of praseodymium oxides were across the surface of the sample, which was confirmed in more detail with higher magnification on the HRTEM image (Fig. 1).

**Figure 3:**
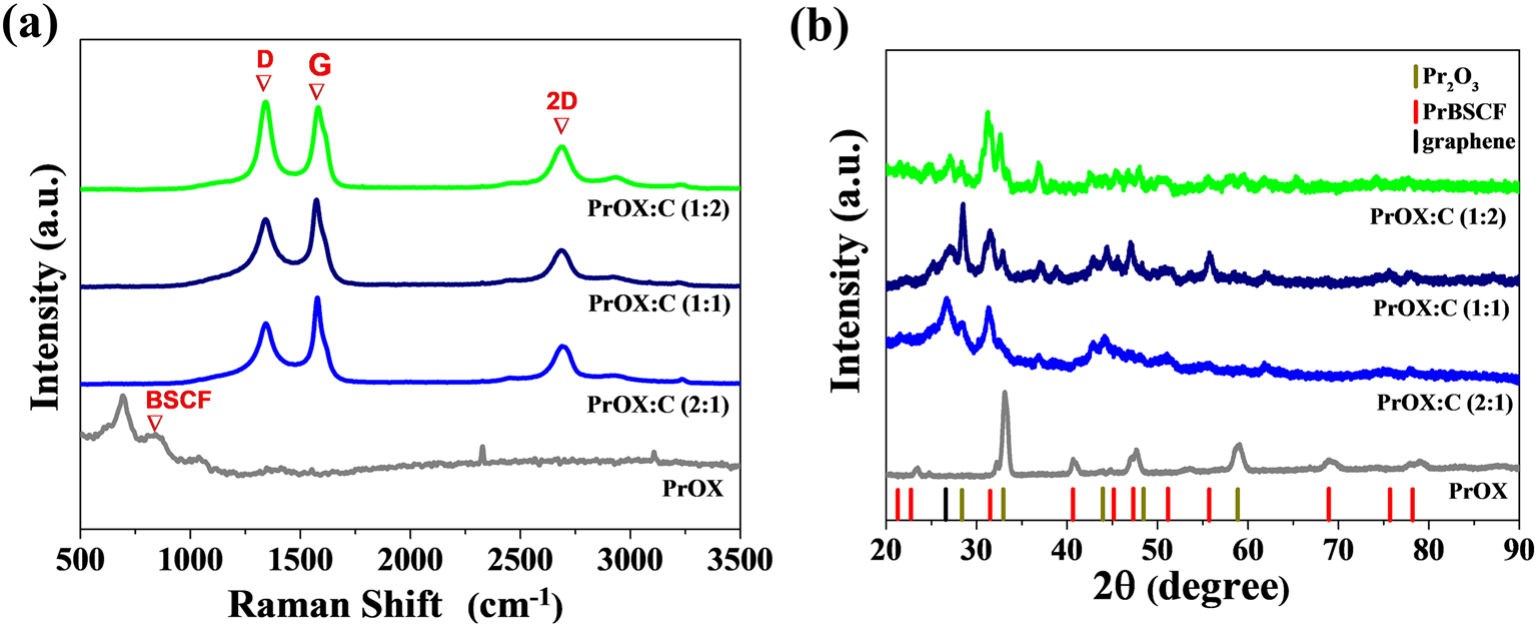
(**a**) Raman spectra and (**b**) X-ray diffraction patterns for PrOX, PrOX:C (1:2), PrOX:C (1:1), and PrOX:C (2:1).

Raman spectroscopy analysis was used to confirm the structure of the materials and the presence of nitrogen-doped graphene in the samples. Raman spectra are provided in Fig. 3a. The spectrum for the PrOX sample has a band at 680 cm^−1^ characteristic of BSCF oxide^9^. For the other samples, no band is visible at 680 cm^−1^, probably due to the presence of bands originating from graphene, which dominates the spectrum with their intensity. Three bands can be observed on the Raman spectrum for the carbon material the D, G, and 2D bands are detailed and analyzed in Table S1. The D band of the presence of free spaces or dislocations in the graphene structure, and the Raman shift of this band is 1350 cm^−1^. The G band indicates the degree of ordering of the carbon network and occurs at 1580 cm^−1^. The 2D band is responsible for the presence of bonds with sp^2^ configuration for graphene and occurs at 2680 cm^−1^^31^. D, G, and 2D bands are present on the spectra of all hybrid samples. To determine the multilayer nature of graphene or the presence of single graphene layers, it is necessary to determine the intensity ratio of the 2D to G bands^32^. Based on an analysis of the spectra of all graphene-containing samples, it can be concluded that the G band is about twice as intense as the 2D band, and a value of the 2D/G band intensity ratio less than 1 indicates the presence of single graphene layers that overlap each other to form multilayer graphene.

The X-ray diffractograms (XRD) presented in Fig. 3b play a crucial role in confirming the formation of perovskite-type materials in our study. These XRD patterns provide valuable insights into the crystalline structure of the synthesized materials. In all samples, including those containing nitrogen-doped carbon, the presence of distinct peaks corresponding to PrBSCF (Praseodymium Barium Strontium Cobalt Ferrite)^9^ and Pr_2_O_3_ (Praseodymium Oxide)^33^ is evident in the XRD data, as indicated by the prominent diffraction peaks at specific angles. These peaks are characteristic of the perovskite-type structure, and their presence confirms the successful synthesis of perovskite-type materials. Furthermore, the XRD patterns of samples that incorporate nitrogen-doped carbon exhibit an additional diffraction line within the range of 25°–27°^34^ This diffraction line is indicative of the presence of a graphene structure within all PrOX:C hybrid materials. This observation underscores the incorporation of nitrogen-doped graphene into the synthesized materials, which enhances their electrochemical properties. Notably, in the case of the PrOX sample, there is no characteristic diffraction peak associated with graphene. This absence of a graphene-related peak confirms that the material consists solely of a mixture of praseodymium oxides without the incorporation of nitrogen-doped graphene.

The nitrogen content of the tested catalysts was confirmed by elemental analysis, the results are summarized in Table S1. It can be observed that the PrOX:C (1:2) sample has the highest percentage of nitrogen content (0.86% by weight). The PrOX:C (2:1) sample has the lowest content of both carbon (44.10% by weight) and nitrogen (0.32% by weight). The results presented here confirm that as the content of carbon material in hybrid structure increases, both carbon and nitrogen content increase.

The surface properties of catalysts are a very important factor affecting electrochemical performance. According to the International Union of Pure and Applied Chemistry (IUPAC), on the basis of pore size, materials can be divided into micropores, which are up to 2 nm in size, mesopores, whose size is in the range of 2–50 nm, and macropores, which are above 50 nm^35^. According to the accepted IUPAC classification^35^, the isotherms obtained (Fig. S2a) are Type II isotherms. Type II isotherms are characteristic of microporous materials. The obtained isotherms also allowed us to calculate the specific surface area of the catalysts using the Brunauer–Emmett–Teller (BET) method, the results are shown in Table S2. The highest specific surface area of 450 m^2^ g^−1^ is characteristic of the PrOX:C (1:2) sample, where the highest amount of added carbon material was used. As one might have assumed, the lowest surface area has the PrOX sample (2 m^2^ g^−1^), which contains no nitrogen-doped carbon in its composition. The pore size distribution curves (Fig. S2b) were determined using the DFT method. It can be concluded that the catalysts studied are characterized by a large number of micropores and the presence of small mesopores. The PrOX:C (1:2) sample has the highest total pore volume and this sample simultaneously has the highest specific surface area (Table S2).

**Figure 4:**
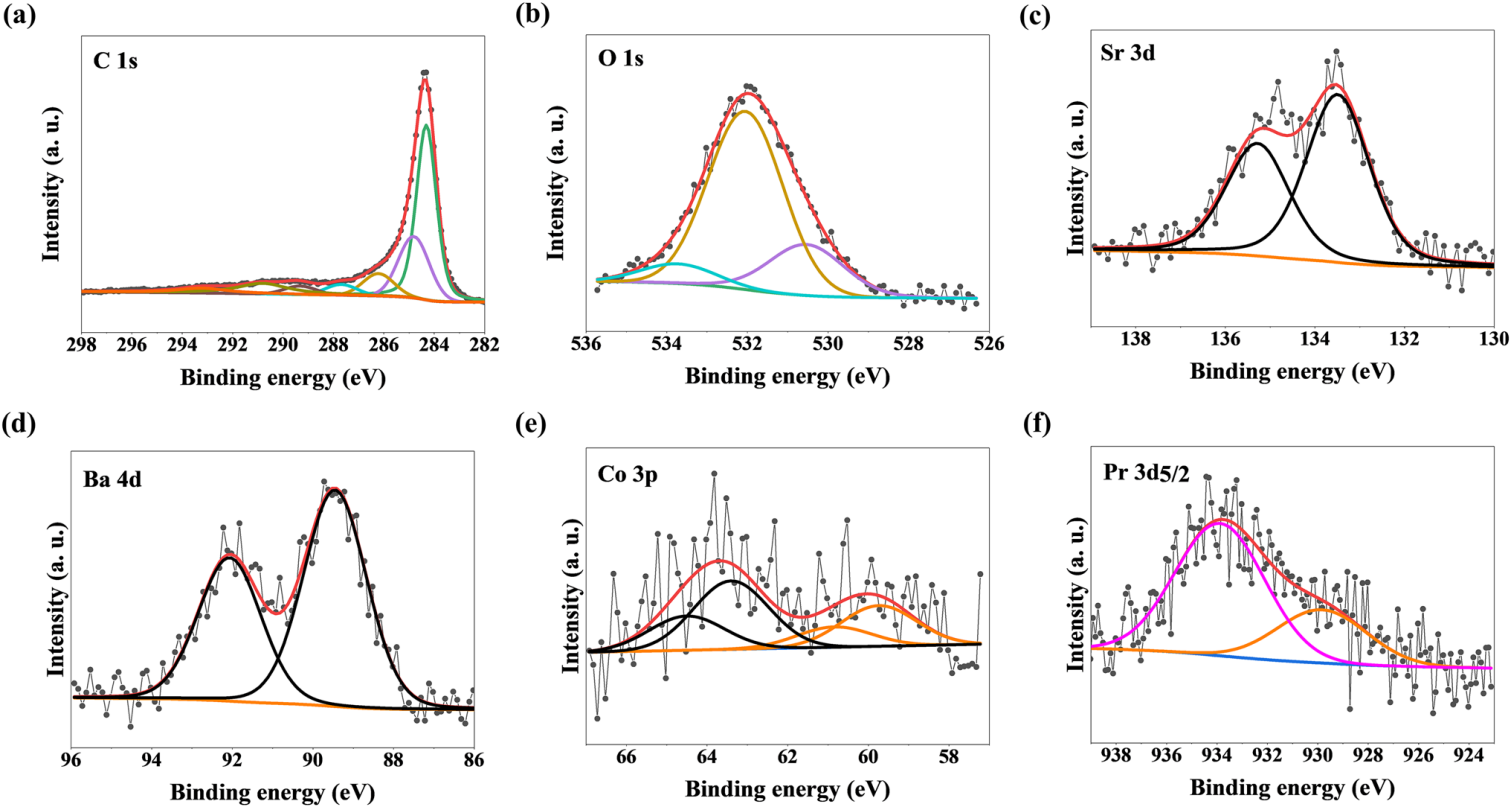
High-resolution XPS spectra of C, O, Sr, Ba, Co, and Pr for PrOX:C (1:2) sample.

In order to determine the elemental composition and chemical state of various elements in the PrOX sample and for PrOX:C series X-ray photoelectron spectroscopy (XPS) measurements were carried out. The survey spectra (Fig. S3) provide the existence of C, Pr, Co, Sr, Ba, and O. From XPS data, the atomic concentration was estimated and detailedescribeded in Table S3. Figure 4 shows high-resolution XPS spectra for the PrOX:C sample (1:2). It can be seen that C1s spectrum for the PrOX:C sample (1:2) can be deconvoluted to seven peaks, but two of them are strong peaks, the strong peaks with binding energies of 284.5 eV and 285.0 eV correspond to C=C sp^2^^36^ and C–C sp^3^ ^36,37^, respectively. The line with an energy of 286.2 eV corresponds to the presence of C–O–C and/or C–OH-type bonds^36,37^, the line lying at an energy of 287.7 eV corresponds to C=O and/or O–C–O bonds^36,37^, the line with an energy of 289.3 eV corresponds to O–C=O-type bonds^36^, and the lines lying at bond energies of 290.8 eV and 293.2 eV, respectively, associated with shake-up excitation^38^. Shake-up excitation originates from sp^2^-type carbon and its aromatic forms and is an additional parameter confirming the presence of this type of bond^36,38^. The O1s spectrum can be fitted into four peaks, the lower binding energy at 528.8 eV is a typical metal–oxygen bonds of Pr − O and Ba − O, the higher binding energy at 530.5 eV of the O1s spectrum is a typical metal–oxygen bonds of O–Me (where Me = Co, Mo, Sr), line at 231.8 eV is assigned to metal–oxygen bonds of O–Me and O=C, line at the higher binding energy 533.2 eV is assigned to O − C or –OH^39,40^. The XPS spectrum of Pr3d_5/2_ can be deconvoluted into two peaks, one at 928.9 eV assigned to Pr^0^ and stronger peak at 933.2 eV, correspond to Pr^3+^^40,41^. The Sr3d spectrum exhibits two different peaks, one at binding energy of 132.1 eV indicating the presence of metallic strontium and the second line lying at a binding energy of 133.7 eV indicating the presence of strontium oxide SrO^40^. The Co3p spectrum exhibits two peaks one at 60.4 eV could be assigned to cobalt in cobalt oxide CoO and second at 60.3 eV correspond to Co^3+^^40^. The Ba4d spectrum posess also two peaks, the first lying at a binding energy of 87.5 eV which indicates the presence of barium oxide BaO, and the second line lying at a binding energy of 90.2 eV which indicates the presence of barium metal^40^.

### Electrochemical results

**Figure 5:**
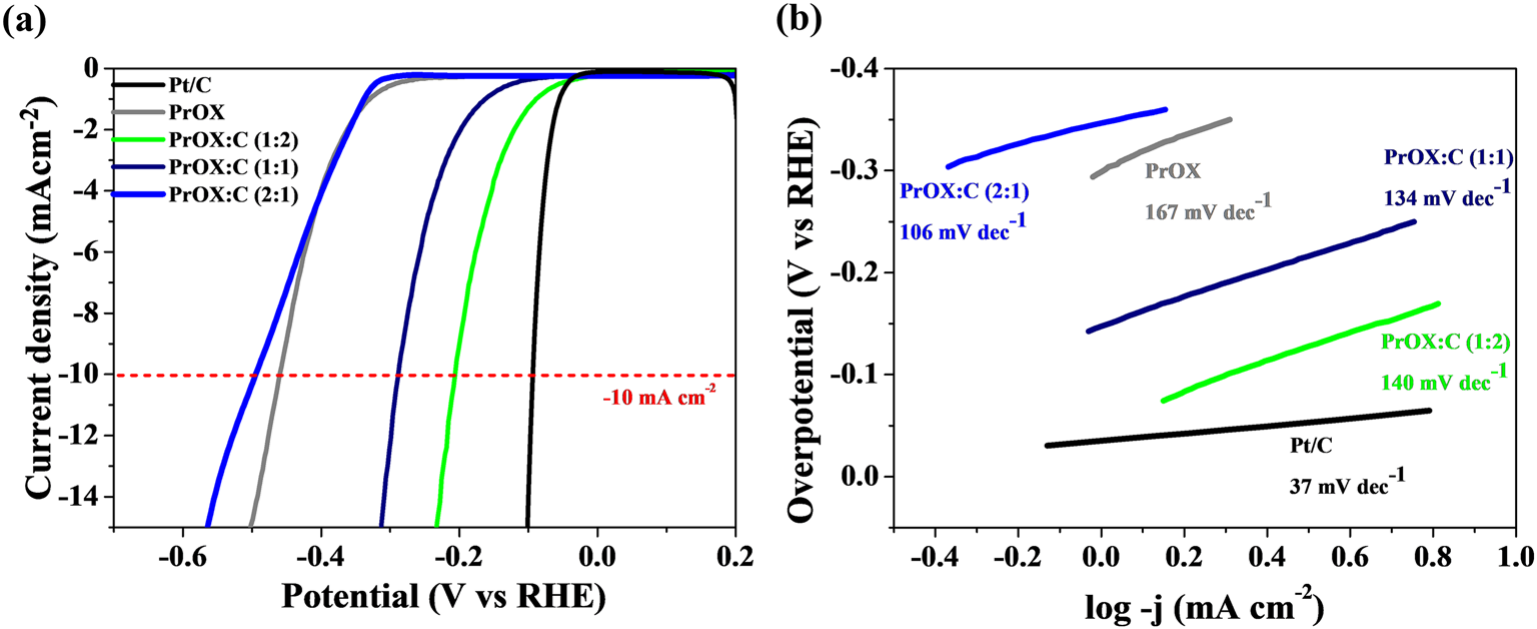
(**a**) The linear sweep voltammetry (LSV) curves measured in 1 M KOH; (**b**) Tafel plots determined from HER LSV curves of the samples.

The hydrogen evolution reaction (HER) tests were conducted on the synthesized pure perovskite and perovskite/carbon materials catalysts in alkaline electrolyte. In Fig. 5a, the linear sweep voltammetry (LSV) curves of the obtained materials, along with commercial Pt/C, are displayed. The perovskite/C hybrid catalysts demonstrate superior HER activity compared to the catalyst without carbon. The addition of carbon to metal oxides has been reported to enhance the hydrogen evolution reaction (HER) activity. This improvement can be attributed to several factors, which have been extensively studied in the literature. Carbon, particularly in the form of carbon nanomaterials such as graphene or carbon nanotubes, possesses excellent electrical conductivity. Incorporating carbon into metal oxides improves the overall electrical conductivity of the composite material, facilitating faster electron transfer during the HER process^42,43^. Carbon-based materials, with their high surface area and porous structure, provide additional sites for catalytic activity. This increases the available active surface area for HER, allowing more efficient electrochemical reactions to take place^44–46^. Moreover, the combination of metal oxides and carbon can result in synergistic effects, where the two components work cooperatively to enhance the overall catalytic performance. Carbon can act as a promoter, facilitating charge transfer and adsorption of reaction intermediates, while metal oxides provide the necessary catalytic active sites^47,48^. Based on the data presented in Fig. 5b, the Tafel slopes calculated for the hybrid catalysts fall within the range of 106–140 mV dec^−1^, which is higher than that of the commercial Pt/C catalyst (37 mV dec^−1^), but lower than the catalytic material without carbon.

This suggests that the hybrid catalysts exhibit intermediate HER kinetics compared to the commercial Pt/C and the catalyst without carbon. There are several reasons why the Tafel slope for the hydrogen evolution reaction (HER) is lower for materials containing carbon. The improved reaction kinetics, facilitated proton transfer, enhanced electrochemical surface area, and synergistic effects collectively lead to a more efficient HER process and a reduced Tafel slope^48,49^.

**Figure 6:**
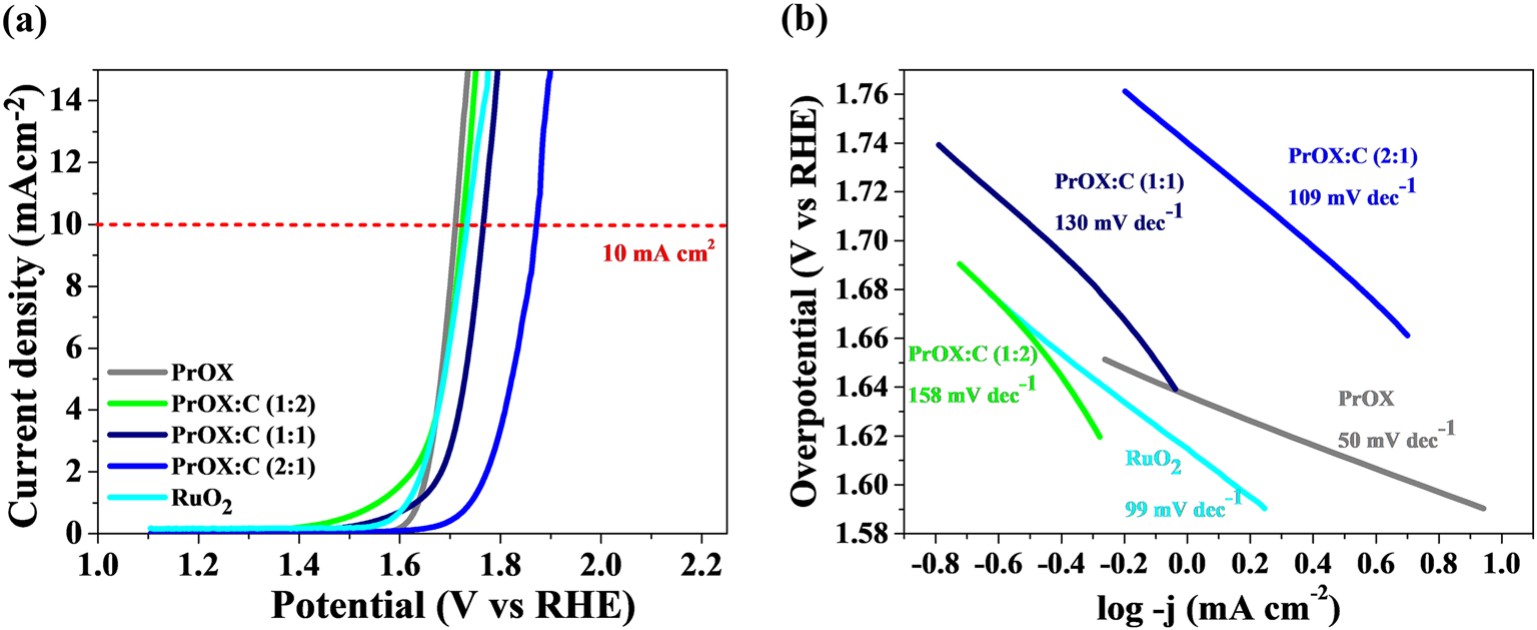
(**a**) The linear sweep voltammetry (LSV) curves measured in 1 M KOH; (**b**) Tafel plots determined from OER LSV curves of the samples.

The OER activity of the synthesized catalyst was investigated in 1 M KOH. In this particular study, the effect of carbon addition on the efficiency of oxygen evolution reaction (OER) was not consistently observed across all materials tested (Fig. 6a). The results did not yield a clear and unequivocal enhancement in OER efficiency upon the incorporation of carbon. It suggests that the presence of carbon does not universally lead to improved catalytic performance for the OER in the investigated materials. The improvement is visible only for the PrOX:C (1:2) material. Moreover, the Tafel slope (only 50 mV dec^−1^) is the lowest for the material without the addition of carbon (Fig. 6b). In some cases, the addition of carbon can lead to an increase in the Tafel slope in the oxygen evolution reaction (OER), which is considered unfavorable. This phenomenon has been investigated in the literature, and several factors contribute to this observation^50^. Especially, in certain conditions, carbon materials can undergo corrosion or oxidation reactions during the OER. The corrosion products or surface modifications can hinder the catalytic activity and result in an increase in the Tafel slope^51,52^.

The electrochemically active surface area (ECSA) was determined to investigate the impact of composition on the HER and OER. The ECSA was estimated using the double-layer capacitance obtained from the charging current at different scan rates using the cyclic voltammetry (CV) method in a KOH solution. The results were plotted in Fig. S4, showing variations in the double-layer capacitance (CDL) depending on the catalyst composition. The ECSA was calculated using a commonly applied specific capacitance value of 0.04 mF cm^−2^. The summarized results can be found in Table S4. However, despite having the highest ECSA, the PrOX (1:1) catalyst did not exhibit superior catalytic properties. Therefore, in this case, it can be concluded that the ECSA has limited influence on the catalytic activity.

The electrochemical stability of materials plays a crucial role in determining their suitability for commercial applications. The stability test results, as presented in Fig. S5 in the Supplementary Information (SI), indicate that the stability of the hybrid catalysts and pure perovskite did not exhibit significant changes compared to the commercial Pt/C even after 25 h of testing. This suggests that the hybrid catalyst material maintains its stability throughout the electrochemical measurements, indicating its potential for practical applications.

**Figure 7:**
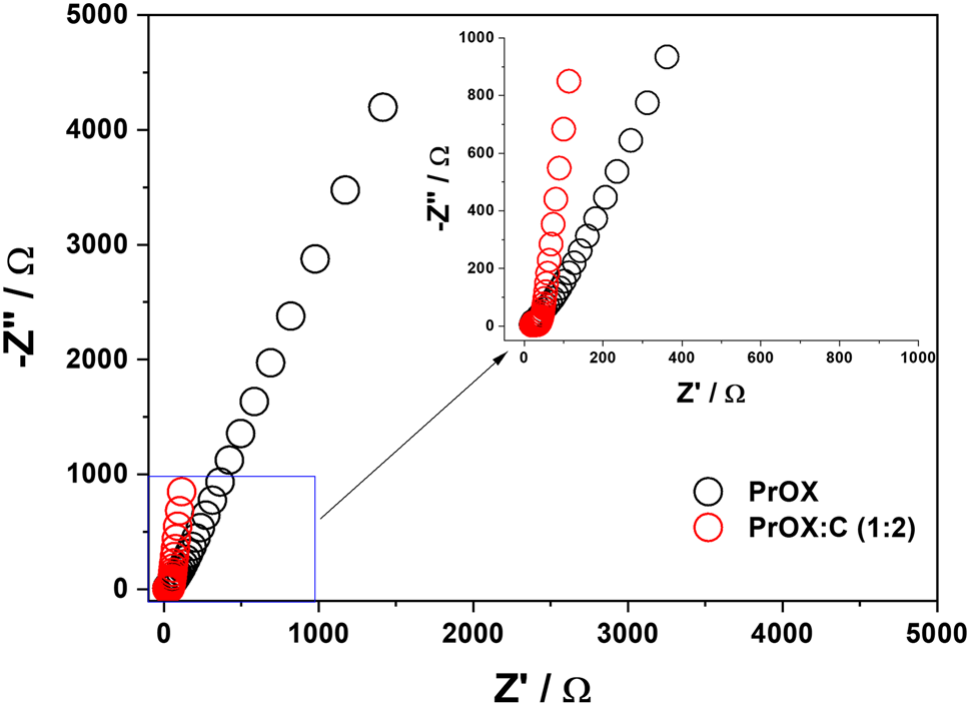
EIS spectra of obtained catalysts for the PrOX and PrOX:C (1:2).

The EIS analysis of the pristine PrOX revealed certain electrochemical properties. The result displayed a higher charge transfer resistance in the EIS curve (Fig. 7). This higher value indicated a relatively lower electron transfer rate within the material, suggesting limited electrocatalytic activity. In contrast, the EIS analysis of the PrOX:C (1:2) showed distinct electrochemical characteristics.

The plot for PrOX:C exhibited a significantly lower charge transfer resistance compared to PrOX, indicating a substantially improved electron transfer rate. This observation strongly suggests enhanced electrocatalytic activity in PrOX:C. Furthermore, the Nyquist plot for PrOX:C displayed a steeper slope, indicating a more capacitive behavior. The EIS results clearly demonstrate a notable improvement in the electron transfer property upon the addition of carbon to the Praseodymium Oxide matrix. The lower charge transfer resistance observed in PrOX:C signifies a more efficient electron transfer process, which is crucial for catalytic applications such as the HER and OER. Additionally, the steeper slope in the Nyquist plot for PrOX:C suggests a more capacitive character, indicating an enhanced electrochemical surface area and accessibility of active sites.

## Conclusions

In this work, a perovskite-type oxide containing praseodymium, barium, strontium, cobalt, and iron atoms dispersed in a carbon matrix was used as a catalyst for hydrogen and oxygen evolution by electrochemical reaction. Compared hybrid material with pristine PrOX oxide demonstrated considerable enhancement in catalytic performance mainly for HER, which can be used as an efficient catalyst for water splitting. The activity can be further promoted by coupling nitrogen-doping and graphene to enhance the activity. The new approach combines elements whose synergistic action contributes to favorable catalytic properties. Also new is the use of porous carbon as an effective matrix for the dispersion of perovskite-type oxide crystallites in their mass. The study contributes to providing a strategy to prepare active and stable noble metal-free catalysts for green hydrogen production.

## Materials and methods

### Synthesis of praseodymium oxide (PrOX)

Firstly, 0.09 g of praseodymium (III) nitrate, 0.03 g of barium nitrate, 0.02 g of strontium nitrate, 0.09 g of cobalt (II) nitrate, and 0.03 g of iron (III) nitrate were placed in a beaker. Then a solution was poured into the solid mixture, which was prepared by mixing 0.30 g of EDTA and 0.34 g of citric acid in 20 cm^3^ of distilled water. To the solution thus prepared, an ammonia solution of 0.5 cm^3^ was added to make the pH of the solution equal to 6. The entire mixture containing the reactants suitable for forming the oxide along with the carbon material was then stirred for a period of 6 h at room temperature. The resulting mass was transferred to a porcelain boat. The mixture was then preheated at 250 °C for a period of 5 h in an inert gas atmosphere, and then at 900 °C for another 5 h. The final product was denoted as PrOX and it is a mixture of two oxides the PrBSCF (Praseodymium Barium Strontium Cobalt Ferrite) and Pr_2_O_3_ (Praseodymium Oxide).

### Synthesis of nitrogen-doped carbon material (C)

The nitrogen-doped carbon material (C) was prepared by mixing 0.24 g of seaweed powder, 0.24 g of graphene nanoplatelets, and 2.64 g of potassium carbonate in 24 cm^3^ of distilled water, respectively, then placed in a laboratory dryer at 110 °C for 1 day to evaporate the water, followed by carbonization at 800 °C with a heating rate of 10 °C/min and annealing at the set temperature for 1 h. From the resulting mass after annealing in an atmosphere of inert nitrogen gas with a purity of 5.0 (99.999%), potassium carbonate is removed with inorganic acid using 6 ml of acid per 0.5 g of carbon, left on a magnetic stirrer for a period of 30 min, after which it is washed with distilled water to obtain an inert pH of the leachate and dried in an electric dryer for a period of 1 day at 110 °C.

### Synthesis of hybrid nanostructures (PrOX:C)

In the synthesis of hybrid nanostructures (PrOX:C), the carbon material was added during preparation PrOX to the reagents after EDTA and citric acid. The abount of used nitrogen-doped carbon material was 0.52 g, 0.26 g or 0.13 g. The hybrid nanostructures based on PrOX and C were obtained by three different mas ratios of reagents 1:2, 1:1, 2:1, then the materials were annealed the same way as PrOX. The obtained samples were labeled PrOX:C (1:2), PrOX:C (1:1), and PrOX:C (2:1).

### Material characterization

High-resolution transmission electron microscopy images and energy dispersive X-ray spectroscopy analysis were manufactured on FEI Europe, model Tecnai F20 X-Twin operated at 200 kV and Quantax 200 with XFlash 4010 detector by Bruker AXS operated at 28 kV. X-ray powder diffraction patterns of samples were carried out by Philips X’Pert diffractometer, which was radiated by graphite monochromatized Cu Kα (λ equal 1.540598 Å). The operating voltage was maintained at 40 kV, the current was maintained at 30 mA and analyzed in the range from 20° to 90°. The X-ray photoemission spectroscopy was performed on PHI5000 PHI VersaProbeII spectrometer using focused monochromatic X-ray of the Al Kα line (1486.6 eV). The vacuum value during the measurement oscillated around 3 × 10^−9^ mbar. The elemental composition (C, N, H) was carried out using a Vario MACRO elemental analyzer from CHN ELEMENTAR Analysensysteme GmbH. Raman spectra were performed on Senterra spectrometer by Bruker Optik. N_2_ adsorption isotherms were carried out on ASAP 2020 Plus instrument (Micromeritics). The specific surface area and pore size distribution curves were determined by the Brunauer–Emmett–Teller (BET) method and density functional theory (DFT).

### Electrochemical measurements

All electrochemical measurements were carried out using a potentiostat–galvanostat (Autolab PGSTAT128). A conventional three-electrode system, consisting of obtained materials on the glassy-carbon (diameter of 3 mm) as the working electrode, a saturated Ag/AgCl electrode as a reference electrode, and Pt plate was applied as the counter electrode. The catalytic ink was prepared by dispersing 3 mg of the samples in 0.75 mL of distilled water, 0.2 mL of isopropanol, and 0.05 mL of a 5% aqueous Nafion solution. The resulting suspension was sonicated in an ultrasonic bath for 60 min. Subsequently, the prepared catalytic ink was deposited onto the polished surface of a glassy carbon electrode and allowed to dry in an oven at 60 °C for a few minutes. The catalytic activity of reference materials such as Pt/C catalyst (20 wt% Pt) for the hydrogen evolution reaction (HER) and ruthenium (IV) oxide for the OER reaction was also measured. The recorded potential measurements were adjusted with respect to a reversible hydrogen electrode (RHE) as the reference electrode. The catalytic activity of the samples in both the oxygen evolution reaction (OER) and hydrogen evolution reaction (HER) was evaluated using a scan rate of 1 mV s^−1^ in a 1 M KOH aqueous electrolyte. The electrochemically active surface area (ECSA) was determined using the cyclic voltammetry technique. Multiple cyclic voltammetry (CV) scans were performed at various scan rates (10, 20, 50, 75, 100 mV s^−1^) within a non-Faradaic region. The double-layer capacitance (CDL) was obtained by calculating the slope of the linear fit from the CV scans. The ECSA of the electrodes was then calculated using the equation: ECSA = C_DL_/C_s_, where C_s_ represents the specific capacitance. In our analysis, we used a Cs value of 0.040 mF cm^−2^, which is a commonly accepted value^53^. Stability tests were conducted on the most promising samples identified from the linear sweep voltammetry (LSV) measurements. The samples were subjected to a constant potential to achieve a current density of 10 mV s^−1^/cm^2^. In addition, Electrochemical Impedance Spectroscopy (EIS) was conducted for the PrOX and PrOX:C (1:2) within a frequency range from 20 kHz to 1 Hz, utilizing a voltage amplitude of 10 mV while maintaining an open circuit potential.

### Supplementary Information

Supplementary Information.

## Data Availability

The datasets generated and/or analysed during the current study are available in the BRIDGE OF KNOWLEDGE repository (https://mostwiedzy.pl/pl/open-research-data/x-ray-diffraction-spectra-of-nitrogen-doped-carbon-in-hybrid-materials-containing-praseodymium-oxide,821012915815912-0).
