# Supplementary material for: Synergistic effects of nitrogen-doped carbon and praseodymium oxide in electrochemical water splitting
Source: Sci Rep. 2023 Oct 30;13:18632. doi: 10.1038/s41598-023-43774-8 (PMC10616108; doi:10.1038/s41598-023-43774-8)
Supplement: Supplementary file 1 — Supplementary Information. [file 41598_2023_43774_MOESM1_ESM.docx]

**Synergistic Effects of Nitrogen-Doped Carbon and Praseodymium Oxide in Electrochemical Water Splitting**

Patrycja Grabowska ^1^, Mariusz Szkoda ^2,3^, Malgorzata Skorupska ^1^, Jerzy P. Lukaszewicz ^1,4^, Anna Ilnicka ^1,^*

^1^ Faculty of Chemistry, Nicolaus Copernicus University in Torun, Gagarina 7, 87-100 Torun, Poland

^2^ Faculty of Chemistry, Department of Chemistry and Technology of Functional Materials, Gdańsk University of Technology, Narutowicza 11/12, 80-233 Gdańsk, Poland

^3^ Advanced Materials Center, Gdańsk University of Technology, Narutowicza 11/12, 80-233 Gdańsk, Poland

^4^ Centre for Modern Interdisciplinary Technologies, Nicolaus Copernicus University in Torun, Wilenska 4, 87-100 Torun, Poland.

***** Corresponding author. E-mail address: ailnicka@umk.pl

**Table S1.** The ratios of the intensities of G, D, and 2D-bands from the Raman spectra of PrOX:C series.

| **Sample** | **cm^-1^** | **I _D_** | **cm^-1^** | **I _G_** | **cm^-1^** | **I _2D_** | **I_D_/I_G_** | **I_2D_/ I _G_** |
| --- | --- | --- | --- | --- | --- | --- | --- | --- |
| PrOX:C (1:2) | 1342.00 | 1.00 | 1581.00 | 0.94 | 2686.00 | 0.49 | 1.07 | 0.53 |
| PrOX:C (1:1) | 1341.50 | 0.78 | 1573.00 | 1.00 | 2686.00 | 0.43 | 0.78 | 0.43 |
| PrOX:C (2:1) | 1343.00 | 0.71 | 1577.50 | 1.00 | 2689.50 | 0.46 | 0.71 | 0.46 |

**Table S2.** The specific surface area and elemental composition.

| **Sample** | **Elemental composition (wt. %)** | | | **S _BET_**  **(m^2^/g)** |
| --- | --- | --- | --- | --- |
|  | N | C | H |  |
| PrOX | nd* | nd | nd | 2 |
| Carbon | 1.22 | 87.89 | 1.22 | 933 |
| PrOX:C (1:2) | 0.86 | 72.19 | 0.64 | 450 |
| PrOX:C (1:1) | 0.64 | 65.87 | 0.66 | 377 |
| PrOX:C (2:1) | 0.32 | 44.10 | 0.47 | 142 |

*nd – not determined.

**
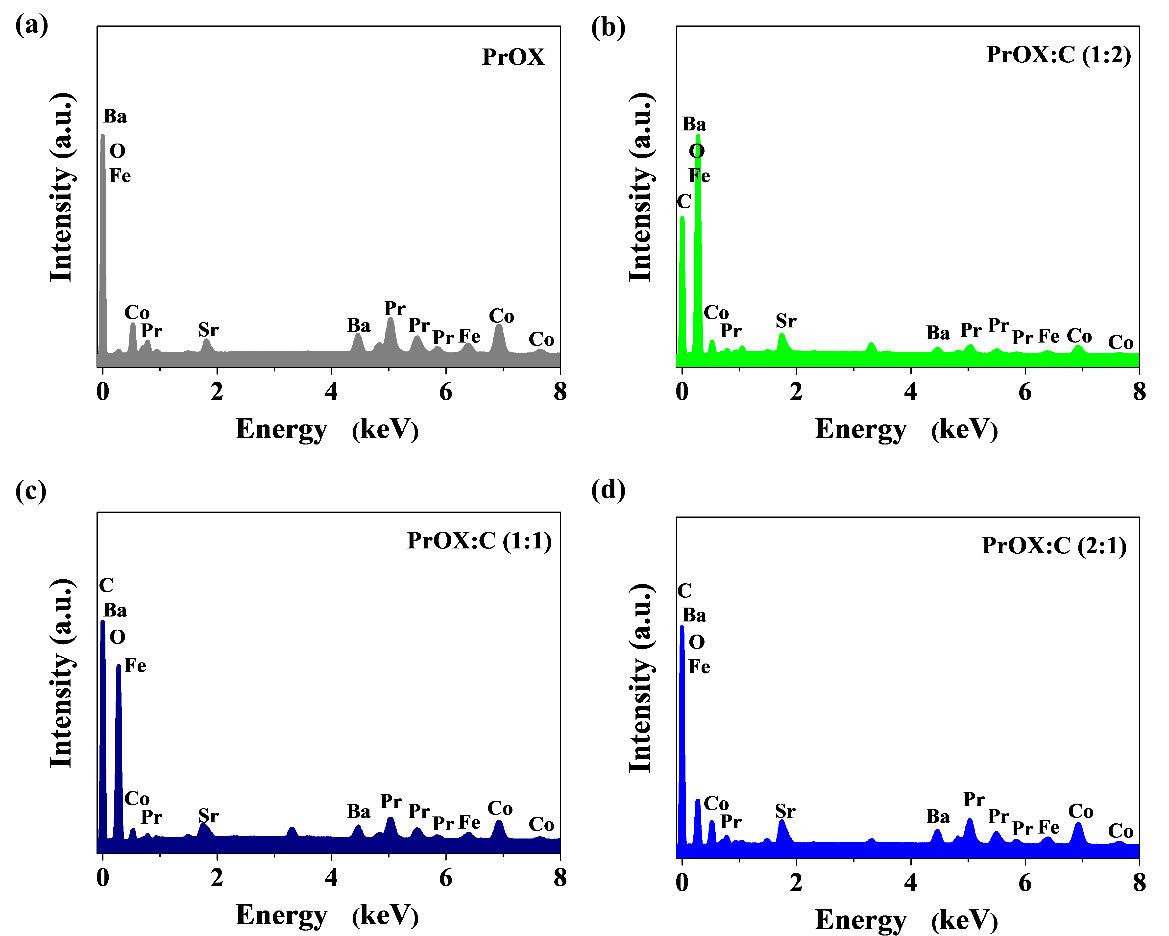
**

**Figure S1.** The EDX profile of (a) PrOX, (b) PrOX:C (1:2), (c) PrOX:C (1:1), (d) PrOX:C (2:1).

**
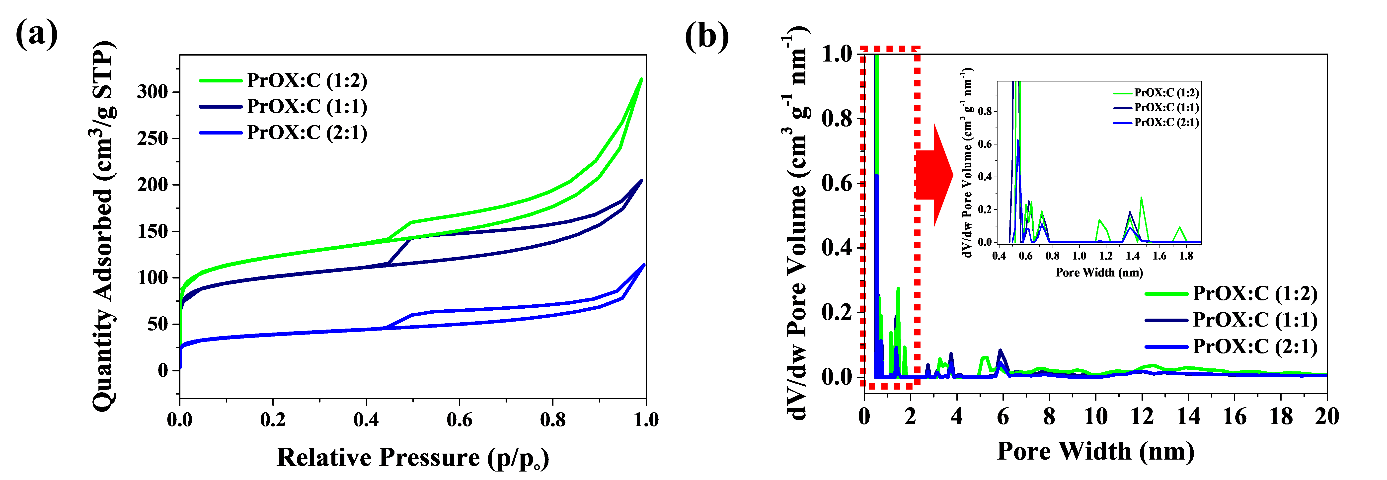
**

**Figure S2.** (a) The comparison of low-temperature (77 K) nitrogen adsorption - desorption isotherms; (b) Pore size distribution curves calculated from the application of the DFT method, insert: zoom of pore size distribution to 2 nm.


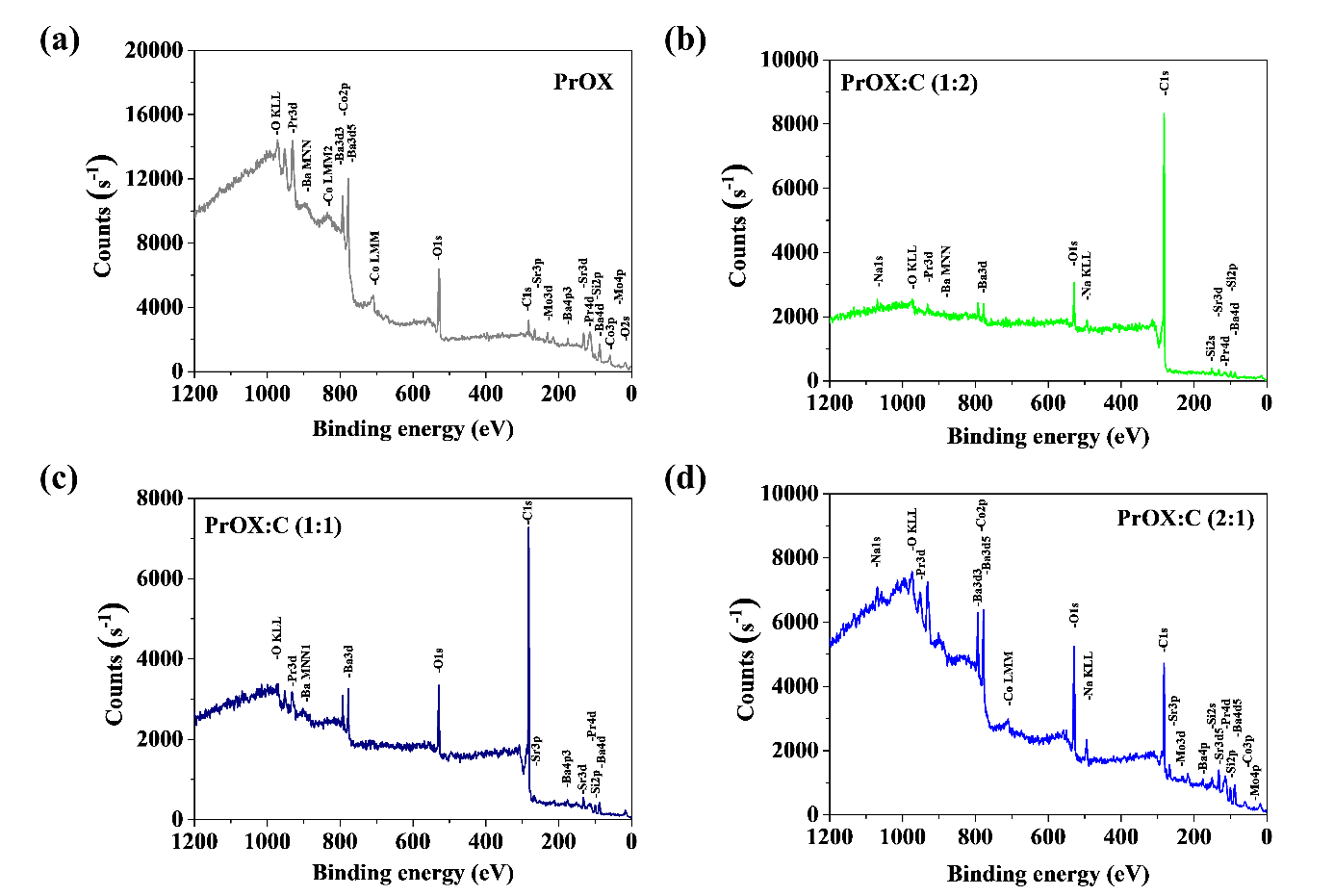


**Figure S3.** XPS survey spectra of **(**a) PrOX, (b) PrOX:C (1:2), (c) PrOX:C (1:1), (d) PrOX:C (2:1).

**Table S3.** XPS determined elements and binding energies.

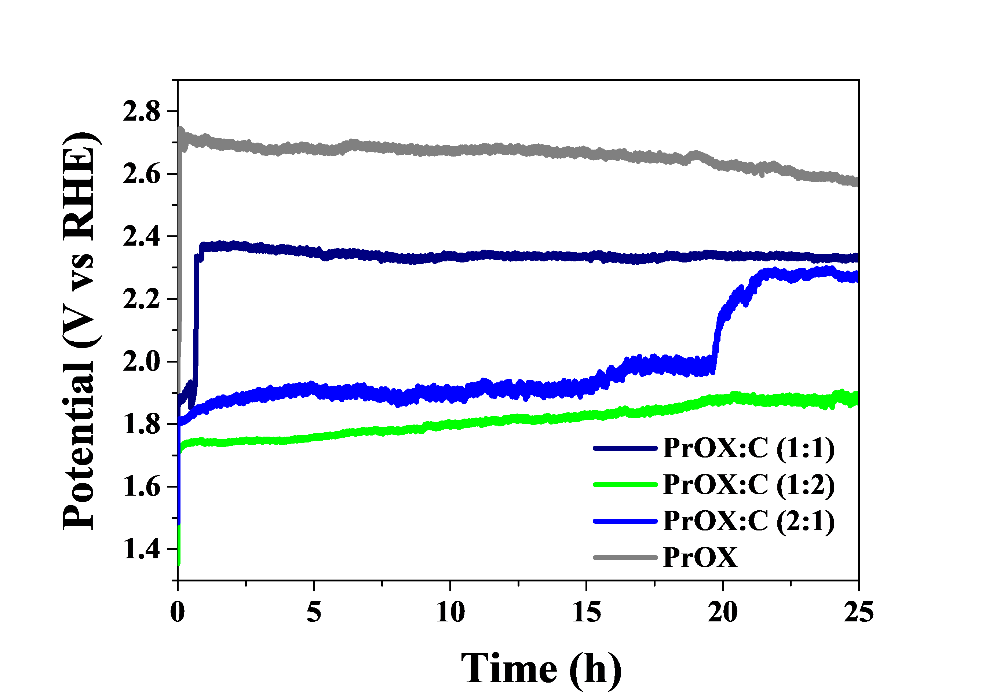


**(b)**

**Figure S4.** Stability of PrOX, PrOX:C series, and Pt/C at a current density of 10 mA cm^−2^ measured in 1M KOH.

**
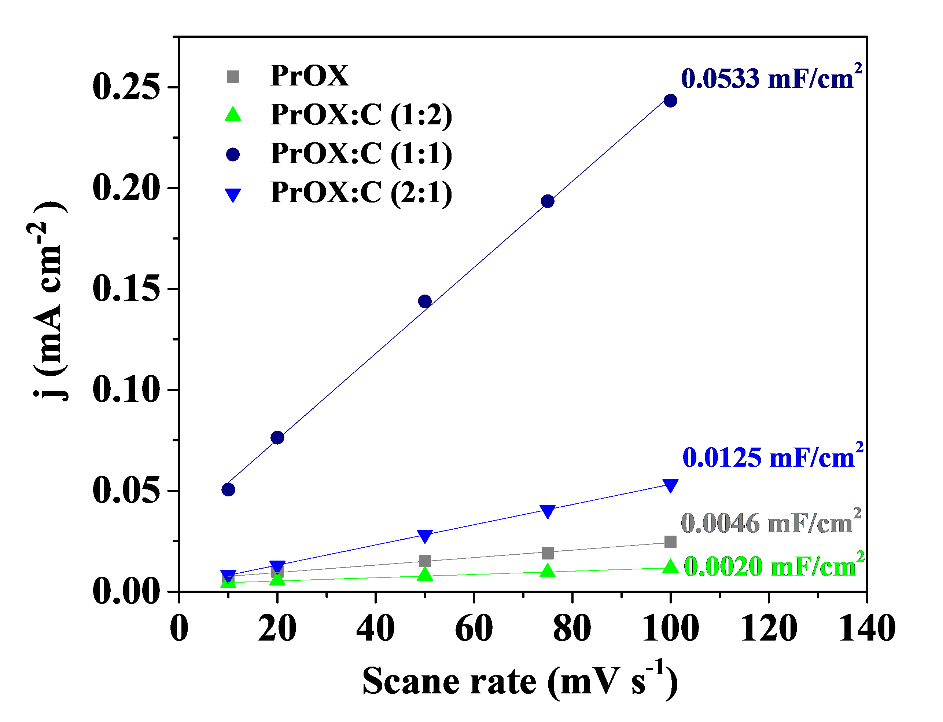
**

**Figure S5.** Calculated double-layer capacitance (CDL) used to estimate the electrochemically active surface area of the obtained catalysts.

**Table S4.** The ECSA parameters for the obtained catalysts PrOX and PrOX:C series.

| **Electrode** | **Estimated ECSA (cm^2^)**  **(0.04 mF cm^−2^) in 1 M KOH** |
| --- | --- |
| PrOX | 0.12 |
| PrOX:C (1:2) | 0.05 |
| PrOX:C (1:1) | 1.33 |
| PrOX:C (2:1) | 0.31 |
